# 5-Alpha-Reductase 2 Deficiency in a Woman with Primary Amenorrhea

**DOI:** 10.1155/2013/631060

**Published:** 2013-12-07

**Authors:** Nasrollah Maleki, Mohammadreza Kalantar Hormozi, Manouchehr Iranparvar Alamdari, Zahra Tavosi

**Affiliations:** ^1^Department of Internal Medicine, Imam Khomeini Hospital, Ardabil University of Medical Sciences, Ardabil, Iran; ^2^The Persian Gulf Marine Medicine Biotechnology Research Center, Department of Endocrinology, Bushehr University of Medical Sciences, Bushehr, Iran; ^3^Department of Internal Medicine, Shohadaye Khalije Fars Hospital, Bushehr University of Medical Sciences, Bushehr, Iran

## Abstract

Steroid 5-alpha-reductase 2 deficiency is a rare disorder leading to male pseudohermaphroditism, a condition characterized by incomplete differentiation of male genitalia in 46,XY patients. Here, we report a case of a 21-year-old woman from Ardabil who presented with primary amenorrhea, ambiguous genitalia, and lack of breast development. All of the serum hormone profiles were normal except for raised serum total testosterone. Testosterone to DHT ratio (T/DHT) was elevated before (15.72) and further increased after hCG stimulation (32.46). A chromosomal study revealed a 46,XY karyotype. A bilateral gonadectomy, recessive cliteroplasty, urethroplasty, and vaginoplasty were performed and hormonal replacement therapy using estrogen was started. In conclusion, the diagnosis of 5-alpha-reductase 2 deficiency may be suspected in infants with ambiguous genitalia or in adolescents or young adults with the characteristic phenotype and serum hormone profiles.

## 1. Introduction

Steroid 5-alpha-reductase 2 deficiency, a 46,XY disorder of sexual development (DSD) [1], is an autosomal recessive condition in which 46,XY subjects with bilateral testes and normal testosterone formation have impaired virilization during embryogenesis due to defective conversion of testosterone to dihydrotestosterone [2, 3]. Dihydrotestosterone (DHT) is essential for the normal development of male external genitalia. DHT is derived from testosterone (T) by a process catalysed by the membrane-bound steroid 5a-reductase enzyme [4]. The enzyme steroid 5*α*-reductase exists in two isoforms, 5*α*-R type 1 and 5*α*-R type 2, which have different expression patterns. Type 1 isoenzyme is encoded by the SRD5A1 gene located on chromosome 5 and expressed at low levels in the prostate, whereas type 2 isoenzyme is encoded by the SRD5A2 gene that maps on chromosome 2 and is expressed at high levels in the prostate and in many other androgen sensitive tissues [5]. 5*α*-reductase deficiency is caused by mutations in the SRD5A2 gene, whereas the SRD5A1 gene has not been found to be mutated in this disease. Although most individuals with 5*α*-reductase deficiency are identified in the neonatal period because of ambiguous genitalia, some are misdiagnosed with androgen insensitivity syndrome, as they often present with the same clinical phenotype, while others escape recognition completely.

In this report, we demonstrate a case of steroid 5-alpha-reductase 2 deficiency that presented with primary amenorrhea and lack of breast development.

## 2. Case Presentation

**Figure 1:**
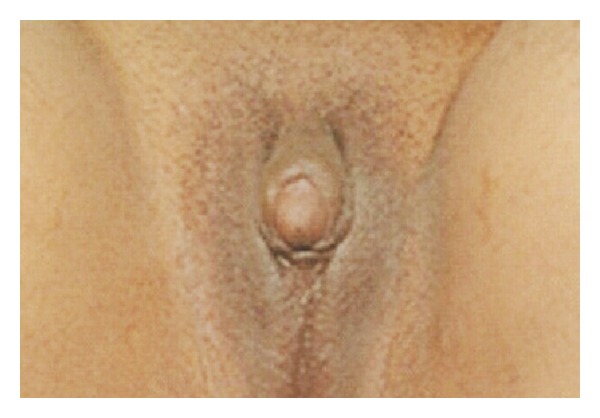
Clitoromegaly and ambiguous genitalia.

**Figure 2:**
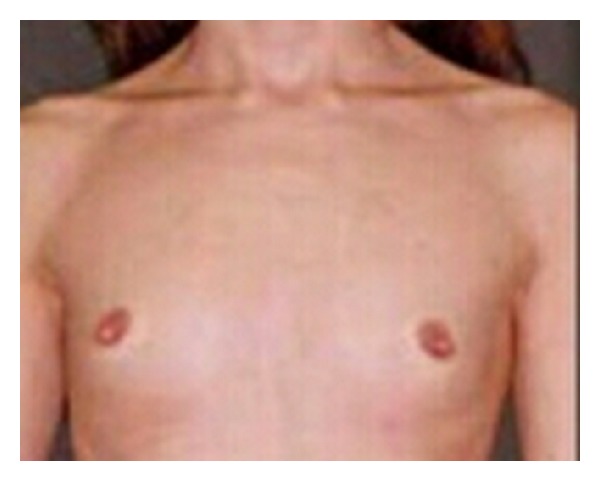
Lack of breast development.

A 21-year-old unmarried woman presented to our medical outpatient department with complaint of primary amenorrhea and lack of breast development. There was no history of significant medical illness in the past. She was born out of a nonconsanguineous marriage. Menstrual history revealed that she never attained menarche. There is no history of significant medical illness in the family. She also had a deep voice. On examination, blood pressure was 110/70 mmHg without significant asymmetry or postural variation with palpable pulses in all extremities. She weighed 71 kg and her height was 174 cm with a body mass index of 23.5 kg/m^2^. General physical examination was within normal limits (no features suggestive of hypothyroidism, Cushing's syndrome, or acromegaly). Her Tanner staging was B2P4A2. External genitalia examination revealed clitoromegaly (4 cm) and ambiguous genitalia (Figures 1 and 2). Gonads were palpable in the inguinal canal bilaterally. She underwent a gynaecological examination which showed a 5 cm blind vaginal pouch. Ultrasonogram of abdomen and pelvis showed atrophic uterus and bilateral ill-defined gonad-like structures high up in pelvic.

Blood investigations showed normal hemogram. Her blood glucose, renal function tests, liver function tests, and serum electrolytes were within normal limits. In view of primary amenorrhea with clitoromegaly, serum total testosterone, LH, FSH, 17-OH Progesterone, DHEAS, thyroid profile, and prolactin were measured. All of the above hormone investigations were normal except for raised serum total testosterone. A chromosomal study revealed a 46,XY karyotype. The diagnosis of 5*α*-reductase deficiency was suspected based on biochemical findings at base line and following hCG stimulation test (hCG 3000 units daily for 3 consecutive days). As shown in Table 1, the patient's testosterone to DHT ratio (T/DHT) was elevated before (15.72) and further increased after hCG stimulation (32.46). The diagnosis of 17*β*-HSD-3, which may have similar clinical presentation, seemed unlikely because of the normal response of androstenedione.

A bilateral gonadectomy, recessive cliteroplasty, urethroplasty, and vaginoplasty were performed and hormonal replacement therapy using estrogen was started.

## 3. Discussion

Steroid 5*α*-reductase 2 deficiency is an autosomal recessive form of male pseudohermaphroditism caused by mutations in the SRD5A2 gene. Over 50 different mutations have been described [5]. The typical clinical features in 46,XY males with 5-alpha-reductase 2 deficiency are those reported in the two originally recognized families as follows [2, 3]. (1) The external genitalia usually are predominately female at birth, with the exception of clitoromegaly in some, so that most affected males are raised as females. A variable degree of virilization occurs at the time of puberty. (2) The internal urogenital tract is male, consisting of epididymis, vasa deferentia, seminal vesicles, and ejaculatory ducts that empty into a blind-ending vagina. The absence of müllerian duct derivatives indicates that anti-müllerian hormone is produced and acts normally. (3) Pubertal gynecomastia may occur, but does not persist into adulthood.

A wide spectrum of phenotypes was reported in a subsequent study of 55 patients with 5-alpha-reductase 2 deficiency including the following [6]: clitoromegaly was present in 27 (49 percent), microphallus in 18 (33 percent), and normal female external genitalia in only four (7.3 percent). 40 patients (72 percent) were initially assigned to female gender, and of these, five (12.5 percent) switched to male sex at puberty.

Concentrations of serum testosterone and estrogens are similar to those in normal men. The level of serum luteinizing hormone (LH) is normal in about one-half of subjects and slightly elevated in the rest. Levels of serum follicle-stimulating hormone (FSH) are elevated. Because of the defect in 5-alpha-reductase 2, the ratio of serum testosterone to dihydrotestosterone is increased, basically in adults and after administration of human chorionic gonadotropin (hCG) in childhood. In the initial descriptions, affected subjects were identified because phenotypic females failed to menstruate and developed variable virilization at adolescence. Now that many affected families have been reported, it is apparent that the manifestations vary [7]. Approximately 55 percent of affected subjects have a blind-ending or pseudovagina in which the Wolffian ducts terminate in the upper vagina. 40 percent of the Wolffian ducts terminate in the perineum on either side of the urethra. The remainder is sufficiently virilized to be assigned male gender at birth. The testes are invariably outside the abdominal cavity either in the inguinal canals, labia majora, or scrotum, and spermatogenesis is impaired.

One report of four individuals diagnosed as adults with intact testes found normal lumbar and hip bone mineral density in three individuals and mild lumbar osteopenia in the fourth [8]. In another study of 16 adult subjects with the disorder, hip bone mineral density was not significantly different than a reference male population [9].

Gender role behavior can change from the female sex of rearing to male at the time of expected puberty. In a large extended kindred from the Dominican Republic, as an example, 18 of 19 subjects initially raised as females subsequently changed gender-role behavior to male at the time of puberty [10]. In other reports, approximately 50 to 60 percent of affected individuals assigned female sex in infancy and virilizing in puberty changed their gender role behavior to male [11]. The percentage of patients changing gender role was much lower (12.5 percent) in the report described above [6], perhaps due to different societal responses to the condition in the different communities studied. Homozygous 46,XX women with 5-alpha-reductase 2 deficiency are phenotypically normal and have normal menstruation and normal fertility, but menarche may be delayed [12, 13].

The diagnosis of 5-alpha-reductase 2 deficiency may be suspected in infants with ambiguous genitalia or in adolescents or young adults with the characteristic phenotype and serum hormone profiles (normal male serum testosterone concentration and increased ratio of serum testosterone to dihydrotestosterone). Measurement of basal concentrations of serum testosterone and dihydrotestosterone is usually sufficient for diagnosis after the expected age of puberty. The ratio of serum testosterone to dihydrotestosterone was >20 in affected subjects in the extended kindred from the Dominican Republic [14] and in another nine unrelated Brazilian families [15]. In the latter study, the ratio ranged from 35 to 84 in affected subjects as compared to 8 to 16 in normal subjects. In adults, measurement of the ratio of serum testosterone to dihydrotestosterone after administration of hCG or testosterone does not increase discrimination between affected and normal men. Definitive diagnosis of the mutation in the steroid 5-alpha-reductase 2 enzyme can be made by cDNA analysis using peripheral blood, biopsy material, or fibroblast cultures [16].

Gonadectomy should be performed to prevent or minimize virilization and to prevent development of tumors in inguinal or labial testes [17]. Clitoromegaly may be surgically corrected by an appropriate procedure to maintain the glans clitoris. If vaginal development is insufficient, vaginal depth should be enhanced by use of dilators at the appropriate age, and, if this is unsuccessful, an artificial vagina should be created surgically [18]. Estrogen therapy that is appropriate for inducing and maintaining feminization should be started at the time of usual pubertal maturation or immediately after gonadectomy is performed in an adult. Such therapy is critical to promote breast development and to prevent osteoporosis. Since virilization is usually less than satisfactory, even when some phallic growth occurs after the time of sexual maturation, trials of supplemental androgen are sometimes undertaken [15, 19]. Although pharmacologic doses of testosterone esters may raise serum dihydrotestosterone concentrations to the normal range and cause acne and growth of facial and body hair, phallic growth is minimal in adults, and the long-term safety of this treatment is not established. The results are different in prepubertal subjects. In one report, as an example, pharmacologic doses of testosterone (two intramuscular doses of testosterone ester 125 mg three weeks apart) led to more than a doubling of penis size [15]. An alternate approach is to administer dihydrotestosterone itself, either in the form of a transdermal cream [20] or an injectable ester [21]. Both modes of dihydrotestosterone administration raise serum dihydrotestosterone concentrations [20, 21], but neither preparation is commercially available, and an optimal regimen has not been established.

Based on the findings of the present case, it is highly recommended that patients presenting with primary amenorrhea, ambiguous genitalia, and lack of breast development perform serum hormone profiles (LH, FSH, 17-OH Progesterone, DHEAS, and hCG stimulation test and measurement of testosterone to DHT ratio) to reveal this rare disorder.

## Figures and Tables

**Table 1:** Results of the hCG stimulation test.

Hormones	Before hCG stimulation test	After hCG stimulation test
Total testosterone (nmol/L)	19.5	39.6
DHT (nmol/L)	1.24	1.22
T/DHT	15.72	32.46
Androstenedione (nmol/L)	6.8	6.5
DHEAS (nmol/L)	4535.2	5435.5
